# White Paper: An Integrated Perspective on the Causes of Hypometric Metabolic Scaling in Animals

**DOI:** 10.1093/icb/icac136

**Published:** 2022-08-06

**Authors:** Jon F Harrison, Andrew Biewener, Joanna R Bernhardt, Joseph R Burger, James H Brown, Zach N Coto, Meghan E Duell, Michael Lynch, Emma R Moffett, Tommy Norin, Amanda K Pettersen, Felisa A Smith, Ummat Somjee, James F A Traniello, Terrie M Williams

**Affiliations:** School of Life Sciences, Arizona State University, Tempe, AZ 85287-4501, USA; Department of Organismic and Evolutionary Biology, Harvard University, Cambridge, MA 02138, USA; Department of Zoology, University of British Columbia, Vancouver, BC V6T 1Z4, Canada; Yale Institute for Biospheric Studies, New Haven, CT 06520, USA; Department of Biology, University of Kentucky, Lexington, KY 40506, USA; Center for Evolutionary and Theoretical Immunology, The University of New Mexico, Albuquerque, NM 87131, USA; Department of Biology, Boston University, Boston, MA 02215, USA; Department of Biology, The University of Western Ontario, London, ON N6A 3K7, Canada; Biodesign Center for Mechanisms of Evolution, Arizona State University, Tempe, AZ 85281, USA; Department of Ecology and Evolution, University of California, Irvine, CA 92697, USA; DTU Aqua | National Institute of Aquatic Resources, Technical University of Denmark, Anker Engelunds Vej 1 Bygning 101A, 2800 Kgs. Lyngby, Denmark; School of Life and Environmental Sciences, The University of Sydney, Sydney, NSW 2006, Australia; Department of Biology, University of New Mexico, Albuquerque, NM 87131, USA; Smithsonian Tropical Research Institute, Panama City, Panama; Smithsonian Tropical Research Institute, Panama City, Panama; Division of Physical and Biological Sciences, University of California, Santa Cruz, CA 95064, USA

## Abstract

Larger animals studied during ontogeny, across populations, or across species, usually have lower mass-specific metabolic rates than smaller animals (hypometric scaling). This pattern is usually observed regardless of physiological state (e.g., basal, resting, field, and maximally active). The scaling of metabolism is usually highly correlated with the scaling of many life-history traits, behaviors, physiological variables, and cellular/molecular properties, making determination of the causation of this pattern challenging. For across-species comparisons of resting and locomoting animals (but less so for across populations or during ontogeny), the mechanisms at the physiological and cellular level are becoming clear. Lower mass-specific metabolic rates of larger species at rest are due to (a) lower contents of expensive tissues (brains, liver, and kidneys), and (b) slower ion leak across membranes at least partially due to membrane composition, with lower ion pump ATPase activities. Lower mass-specific costs of larger species during locomotion are due to lower costs for lower-frequency muscle activity, with slower myosin and Ca^++^ ATPase activities, and likely more elastic energy storage. The evolutionary explanation(s) for hypometric scaling remain(s) highly controversial. One subset of evolutionary hypotheses relies on constraints on larger animals due to changes in geometry with size; for example, lower surface-to-volume ratios of exchange surfaces may constrain nutrient or heat exchange, or lower cross-sectional areas of muscles and tendons relative to body mass ratios would make larger animals more fragile without compensation. Another subset of hypotheses suggests that hypometric scaling arises from biotic interactions and correlated selection, with larger animals experiencing less selection for mass-specific growth or neurolocomotor performance. An additional third type of explanation comes from population genetics. Larger animals with their lower effective population sizes and subsequent less effective selection relative to drift may have more deleterious mutations, reducing maximal performance and metabolic rates. Resolving the evolutionary explanation for the hypometric scaling of metabolism and associated variables is a major challenge for organismal and evolutionary biology. To aid progress, we identify some variation in terminology use that has impeded cross-field conversations on scaling. We also suggest that promising directions for the field to move forward include (1) studies examining the linkages between ontogenetic, population-level, and cross-species allometries; (2) studies linking scaling to ecological or phylogenetic context; (3) studies that consider multiple, possibly interacting hypotheses; and (4) obtaining better field data for metabolic rates and the life history correlates of metabolic rate such as lifespan, growth rate, and reproduction.

## General introduction to hypometric metabolic scaling

“It is only a slight overestimate to say that the most important attribute of an animal, both physiologically and ecologically, is its size. Size constrains virtually every aspect of structure and function and strongly influences the nature of most inter- and intraspecific interactions. Body mass, which in any given taxon is a close correlate of size, is the most widely useful predictor of physiological rates.” (Bartholomew GA. 1981. A matter of size: an examination of endothermy in insects and terrestrial vertebrates. In: Heinrich B, editor. Insect thermoregulation. New York (NY): Wiley. p. 45–78.)

Of the many fundamental ecological and life-history traits that scale with body mass, the relationship between aerobic metabolic rate and body size has been particularly well characterized, but arguably, never explained. In 1933, Max Kleiber demonstrated that whole-organism resting metabolic rates (MRs) of mammals and birds scale with approximately the 3/4 power of body mass (BM), such that MR = *a*BM*^b^*, with *a* being the intercept of the regression line, and the scaling exponent, *b*, being 0.75. This means that the relationship is hypometric with larger animals expending less energy per gram of body mass than smaller ones. Hypometric scaling of metabolic rate also occurs within ectothermic animals, protists, and plants (Hatton et al. 2019). Large and small animals differ in many other characteristics, with larger animals consuming food at a lower rate per gram, and having relatively more muscle but smaller brains, more fat, slower relative running speeds, slower limb cycling frequencies, and longer lifespans than smaller-bodied ones, to name just a few of the many correlates of body size (Table 1). While these empirical patterns have been recognized for at least a century, the underlying causal mechanisms remain highly controversial (e.g., Rubner 1883; McMahon et al. 1983; Peters 1983; Schmidt-Nielsen 1995; Calder 1996; West et al. 1997; Glazier 2005; Glazier 2010; Sibly et al. 2012; Hatton et al. 2019).

**Table 1:** Relative values for some morphological and physiological parameters in an actual 10 kg mammal (predicted from allometric equations in the cited study) compared to a 10 g mammal isometrically scaled to 10 kg.

Parameter	Value compared to isometric prediction	Reference
Heart mass, g	1	(Prothero 2015)
Muscle mass, g	1.6	(Alexander 1981)
Brain mass, g	0.06	(Tsuboi et al. 2018; Burger et al. 2019)
Fat mass, g	4	(Schoenemann 2004)
Mass-specific resting metabolic rate, watts	0.12	(White et al. 2009)
Mass-specific food consumption rate, g s^-1^	0.20	(Nagy 2001)
Running speed, body lengths s^-1^	0.73	(Iriarte-Diaz 2002)
Stride frequency, movements s^-1^	0.35	(Heglund and Taylor 1988)
Lifespan, s	4.3	(Speakman 2005)

Among these parameters, only heart mass scales isometrically, being the same relative value in large and small mammals. Usually similar patterns, though with less complete data, are observed for many taxa, and across other size ranges.

This manuscript is written by participants in a SICB symposium titled “Causal mechanisms of metabolic scaling.” The focus of this symposium is to bring together researchers with diverse perspectives working on multiple aspects of metabolic scaling to increase our understanding of the patterns and processes. The goal of this “white paper” is for the symposium participants to work together to integrate their perspectives by agreeing on terminology, possible hypotheses to explain observed patterns, and pointing toward research directions we mutually agree to be worthwhile. As for multiple other very important questions in biology, explanations for hypometric metabolic scaling are unresolved, and we hope that this paper will aid interested scientists in the development of an agreed-upon theory. Doing so requires that participants in the field directly address each other's arguments, recognize the context-dependence of biological processes, and consider multiple hypotheses (Norberg et al. 2022). We do not attempt to review all the evidence for and against the various hypotheses, and refer readers to excellent, more comprehensive reviews on this subject (Glazier 2014; White and Kearney 2014; 2018; Harrison 2018; Kozłowski et al. 2020). While the focus of this white paper is on animals, we believe that most of the points made are likely generally applicable to the majority of organisms.

As for many still-unresolved-topics in biology, the answer to how and why metabolic rates scale hypometrically will depend in part on how the question is framed: Are empirical patterns consistent or are they highly variable? To the extent that metabolic scaling is variable, how and why does this variation reflect the phylogeny and environment? With respect to causation, what is the relation between the universal constraints (i.e., laws of physics and first principles of biology) and the vagaries of biodiversity (i.e., enormous variety of form, function, behavior, and ecological interactions)? To what extent does the scaling of metabolic rates reflect the role of natural selection in shaping the evolution of other anatomical, physiological, behavioral, life-history, and ecological traits? Are the deviations from overall scaling relations for taxonomic and functional groups consistent with the causal hypotheses—that is, exceptions that prove the rule—or are they idiosyncrasies that require different theoretical foundations?

## Scaling terminology

The pattern of larger animals using less energy per gram has been variously termed negative allometric scaling and hypometric scaling. The term “negative allometry” is applied when log mass-specific parameters plotted vs. log mass exhibit a negative linear slope. Statistical analyses of relationships in which the same parameter (in this case, mass) are included in both the dependent and independent parameter are problematic (Packard and Boardman 1989; Raubenheimer 1995). Therefore, throughout this manuscript, we use the term hypometric scaling when exponents are statistically less than the predicted values for isometric scaling (i.e., when *b* < 1 for volumes), and the term hypermetric scaling when these exponents are statistically greater than the isometric prediction (*b* > 1 for volumes). This terminology requires that we define the isometric condition. Isometric scaling refers to the situation in which a larger organism is simply a larger but otherwise identically shaped, formed, and functioning organism (*b* = 1).

For morphology, isometry is well-defined and noncontroversial, assuming equivalent geometric shapes. Dating back to at least Archimedes, it was established that surface areas increase with the square of the length, and volume with the cube. For isometric scaling, on log–log plots, volumes and masses scale with mass^1^, areas with mass^0.67^, and lengths with mass^0.33^.

The isometric predictions for rates and durations are more problematic and controversial. A. V. Hill (Hill 1950), using classical dimensional analysis, argued that isometrically scaled animals of different size should run at similar speeds, requiring faster limb cycle frequencies in smaller animals, scaling with mass^–0.33^ and with stride lengths expected to scale with mass^0.33^. However, from a biological perspective, we know that to contract at different speeds, muscles require different properties. Faster muscles generally have higher contents of fast-type fibers, with faster myosin ATPases, more sarcoplasmic reticulum, and different Ca^++^ATPases and troponin regulatory proteins. Thus, if a larger animal was simply a scaled-up version of a smaller animal (with identical muscles, fiber types, and myosin ATPases), the isometric prediction would be that limb cycle frequency should be invariant, stride length should scale with mass^0.33^, and speed with mass^0.33^. In actuality, limb cycle frequency in quadrupedal mammals scales with mass^–0.15^, and large animals tend to run absolutely faster, but slower when normalized to body length (Biewener and Patek 2018). If our perspective is from classic dimensional analysis, larger animals have faster limb frequencies and speeds than predicted (hypermetric scaling), but from a biological perspective, larger animals have slower limb cycle frequencies and speeds than if they were simply scaled-up versions of smaller forms (hypometric scaling).

A related confusion exists between classical dimensional analysis and the biological perspective when considering durations. As noted above, biomechanists have usually considered that with isometric scaling, times such as limb cycle frequencies should scale with mass^–0.33^, and so durations scale with mass^0.33^. Lifespans have been empirically shown to scale with mass^0.25^, so near to the isometric prediction for classical dimensional analysis (Lindstedt and Calder 1981; Hatton et al. 2019). However, if our perspective is that larger, isometrically scaled animals should be simply scaled-up versions of small ones, with identical tissue composition, then the isometric prediction should be that lifespan is invariant with size. From this perspective, lifespan scales hypermetrically (exponent greater than the isometric prediction of zero). Supporting this conclusion, larger animals have evolved a variety of cellular adaptations that promote longer lifespan, including more anti-cancer genes, membrane lipids less sensitive to oxidative damage, lower mass-specific metabolic rate, lower accumulation rate of somatic mutations, and lower Reactive Oxygen Species(ROS) generation rate (Hulbert et al. 2007; Seluanov et al. 2008; Blagosklonny 2013; Dang 2015; Cagan et al. 2022). These different perspectives demonstrate that isometry from the perspective of classical dimensional analysis is not the same as thinking of larger animals as otherwise identical, scaled up versions of smaller animals. Thus, the best use of these terms will depend on the question posed.

Most authors have suggested that the isometric prediction for rate processes such as metabolic rate and food consumption rate is for these to scale with mass^1^, assuming volume is directly proportional to mass and that large and small animals have identical composition. The central question motivating this manuscript and symposium is why aerobic metabolic rates and many associated variables of most animals scale hypometrically rather than isometrically.

Hypometric metabolic scaling can be studied in at least three contexts: (1) during *ontogenetic growth*, (2) *intraspecific variation* among individuals at the same developmental stage within or across populations (often termed static allometry), and (3) *interspecific variation* in body size (sometimes called evolutionary allometry). Studies included in this volume of *ICB* cover each of these various contexts, examining what may be the underlying causal mechanisms for observed patterns of metabolic scaling.

## One causal explanation, or many?

The observation that relatively similar hypometric scaling occurs across diverse clades and body sizes has motivated searches for a single, overarching explanatory theory. In support of a single, universal explanation, scaling slopes for metabolism and the many correlated variables are often quite similar across clades and physiological states (Peters 1983; Savage et al. 2004; Hatton et al. 2019; Brown et al. 2022). Given these similarities, a single cause would be the most parsimonious explanation.

Yet, diversity is the hallmark of biology. Since Kleiber's seminal work (Kleiber 1947), many hypotheses have been proposed for the hypometric scaling of metabolism and the associated changes in life history, behavior, anatomy, physiology, and cell and molecular biology (Table 2; [McMahon et al. 1983; Peters 1983; Schmidt-Nielsen 1995; Calder 1996; West et al. 1997; West et al. 2000; Glazier 2010; Sibly et al. 2012; 2014; Glazier 2015]). Is there one hypothesis to rule them all, or might hypometric metabolic scaling arise from multiple interacting causal factors, perhaps in a context-dependent way?

**Table 2:** Why and how do larger animals have lower mass-specific metabolic rates?

Why? (Physical, network, population genetic, or biotic causes)		
Category		Sample reference
Geometry	Declining body surface-to-volume ratios reduce mass-specific heat loss and production (homeothermic endotherms)	(Roberts et al. 2010)
	Declining body or gut surface-to-body volume ratios reduce mass-specific nutrient intake Dynamic Energy Budget models(DEB).	(Kooijman 1986)
	Declining bone and muscle area-to-body mass ratios increase fragility, requiring slower movements or changes in locomotory limb posture in terrestrial vertebrates.	(Biewener 2005; Norberg and Aldrin 2010)
	Efficient rates of transport through circulatory systems decline (Fractal MTE theory).	(West et al. 1998)
Social networks	Social synergies/division of labor among components increase efficiency.	(Fewell and Harrison 2016)
Population genetics	Accumulation of negative mutations due to long generations and small populations impair protein function (drift barrier hypothesis).	(Lynch 2022)
Biotic interactions (ecology, correlated selection, and trade-offs)	Trade-offs reduce mass-specific growth and locomotory performance.	(Kozłowski et al. 2020)
	Trade-offs increase capacities to survive poor resource conditions.	(Harrison 2017)
	Need for greater absolute food intake with competition increases selection for efficient use of food.	(Witting 2017)
	Trade-offs reduce mass-specific neural performance.	(Coto and Traniello 2022)
	Sexual selection produces larger, lower cost ornaments/weapons	(Somjee 2022)
**How? (Proximate behavioral, physiological and molecular mechanisms)**		
Systemic (field MR)	Reduced foraging, feeding, digestion, absorption, anabolism, growth, reproduction.	(Peters 1983)
System composition (basal, resting, and field MR)	Reduced content of expensive tissues (brain, sense organs, liver, and kidneys).	(Kozłowski et al. 2020)
	Increased content of inexpensive tissues (fat and bone).	(Schoenemann 2004)
Locomotion-related (field, active, and max MR)	Slower limb cycle frequencies associated with lower myosin ATPase activities.	(Bárány 1967; Heglund and Taylor 1988)
	Increased elastic energy storage.	(Pollock and Shadwick 1994)
Cellular (basal, resting, field, and max MR)	Reduced ion leaks across mitochondrial and cell membranes reduce costs.	(Hulbert et al. 2002)
	Larger cells have reduced ion transport costs (due to lower surface-to-volume ratios)	(Kozłowski et al. 2010)
	Lower maximal protein-specific catalytic rates.	(Turner et al. 2006)

Here, we list and categorize a sample of hypotheses put forward to explain the hypometric scaling of metabolic rate in animals, categorized as answering why vs. how questions. For the “how” questions, we also categorize the hypotheses as to the physiological state for which they are most relevant.

While metabolic rates generally scale hypometrically, many recent authors have concluded that metabolic scaling slopes are not universal, but rather differ with physiological state and clade (White et al. 2007; Glazier 2010; Burgess et al. 2017; Kozłowski et al. 2020). As examples, different metabolic scaling patterns are observed for different clades of mammals (White et al. 2009), scaling exponents tend to be higher among larger mammals (Clarke et al. 2010), and fish exhibit scaling exponents close to one (White et al. 2006; Killen et al. 2016). The observation that metabolic scaling can be influenced by phylogeny supports the idea that the causes of metabolic scaling can be influenced by biological form, ecology, and genetic background. Norin addresses this idea in this volume, examining the possibility that size-selective predation for fast growth can drive steep metabolic scaling in fish (Norin 2022). Alternatively, Brown and co-authors in this volume (Brown et al. 2022) consider the possibility that much of the variation in scaling relations may be due to measurement error (inconsistent definitions of parameters and differences in methodology).

Hypometric scaling of aerobic metabolic rates has commonly been observed for many physiological states, including standard (ectotherm), basal (endotherm), field, and maximal aerobic metabolic rates (Savage et al. 2004). A pattern that has been observed for many clades is that active metabolic rates tend to scale more steeply than resting (Suarez and Darveau 2005; Glazier 2010). Because the use of ATP is very different under these different physiological circumstances, it is certainly plausible that different causal mechanisms might drive scaling patterns during these different activities (metabolic boundaries hypothesis, [Glazier 2010]). For example, during locomotion, ATP use by skeletal muscle predominates, so effects of size on limb and muscle contraction frequency that drive differential use of ATP by myosin and calcium ATPases seem likely to be central in determining metabolic scaling. Whereas, during post-absorptive measurements of standard or basal metabolic rates, ATP use is thought to be primarily associated with processes such as maintenance of ion gradients, protein cycling, gluconeogenesis, renal activity, and basal cardiorespiratory activity. Most of these processes seem more likely to be related to cell activity and perhaps cell size rather than body size, suggesting different causes of hypometric scaling.

Conversely, correlations between metabolic rates might enable a single cause to drive hypometric scaling during all activities. For example, constraints on maximal aerobic metabolic rates in larger animals could select for animals with reduced densities of ATP-using and producing molecules, leading to reduced mass-specific standard or basal metabolic rates. Across species, maximum aerobic metabolic rates are positively correlated with standard or basal metabolic rate in both ecto- and endotherms (Bennett and Ruben 1979; Killen et al. 2016), and this pattern also holds within at least some species (Norin and Malte 2012).

As noted above, hypometric metabolic scaling has been observed during animal ontogeny and across species. Some of the hypotheses for metabolic scaling seem unlikely to apply to both contexts. For example, nuclear and mitochondrial mutation load are unlikely to vary during juvenile ontogeny as they do across species with similarly different body sizes. Transport of fuels across guts with different surface-to-volume ratios or ecological food availability may differentially affect species of different sizes, but seem unlikely to affect embryonic, yolk-fueled growth. Such considerations support the idea that multiple causes may drive metabolic scaling in different contexts.

Hypometric scaling of metabolic rates has often been documented in colonies of social insects and marine invertebrates (Hou et al. 2010; White et al. 2011; Fewell and Harrison 2016; Burgess et al. 2017). Some types of hypotheses for metabolic scaling seem impossible to apply to social insect colonies; for example, when ant colonies are studied in the lab, oxygen and food availability to individuals does not depend on colony size, and surface area and distribution network scaling seems very different. Either hypotheses that depend on such size variation are not universal and multiple causes are required, or we must find causal explanations that can cover this wide array of contexts and organism types.

## The logical framing of causal hypotheses for metabolic scaling

Evolutionary hypotheses for observed patterns are necessarily historical, because these patterns have evolved independently in many lineages, and multi-level, because they imply coevolution of the entire phenotype, from molecules to individuals, from biochemistry and physiology to ecology. Hypotheses for the pattern of hypometric scaling should address both why (ultimate) questions and how (proximate) questions. Ultimate questions focus on the historical, evolutionary, or population-genetic explanations, while proximate questions focus on the behavioral, physiological, cellular, and molecular mechanisms (Table 2). This framing follows Mayr (Mayr 1961), recognizing that evolutionary causes and functional mechanisms necessarily interact (Laland et al. 2011), and that other types of questions are possible (Calcott 2013). Ultimate and proximate questions are not alternative, but together can provide a full explanation.

Dividing the hypotheses for hypometric scaling of metabolism into these two categories is surprisingly challenging. One reason for this is that at levels of societies, populations and ecosystems, behavioral and ecologicical processes necessarily reflect mechanisms at molecular, cellular, and individual levels. Secondly, many of the ultimate hypotheses for metabolic scaling include mechanistic elements. For example, perhaps the most common and oldest answer to the ultimate question blends with proximate hypotheses by focusing on changes in the geometrical dimensions of animals of different sizes (Table 2). As organisms increase in size, the surface-to-volume ratio decreases, scaling with mass^0.67^ for many shapes. These changes alter the rate of heat loss, especially important for endotherms, and potentially affect oxygen, nutrient, waste, ion, and water exchange across body surfaces, and perhaps the gut. Similarly, the social synergies hypothesis, which depends on the concept that systems with more components may be able to increase efficiencies by specialization of components, blends ultimate and proximate considerations. Only the drift barrier hypothesis, and explanations based on biotic interactions fall clearly into the realm of classic evolutionary explanations for this pattern. Some of these geometrically based hypotheses have also been called “constraint hypotheses” as they include the concept that metabolic rates of larger organisms are constrained by a physical factor associated with large size, such as a low surface-to-volume ratio or fragility (Brown et al. 2018; Harrison 2018). This blending of ultimate and proximate mechanisms for many of the prominent hypotheses for hypometric metabolic scaling may provide a partial philosophical explanation for why this topic has been so challenging for biologists to grapple with.

In general, we have much better mechanistic answers to the proximate questions than the ultimate questions for hypometric metabolic scaling, at least for interspecific comparisons. At a physiological level, the scaling of resting metabolic rate may be mostly explained by varying composition of expensive tissues and a lower rate of energy use in tissues of larger animals (Wang et al. 2012; Kozłowski et al. 2020). An important point is the patterns predicted by the various proximate explanations (e.g., larger animals having decreased mass-specific food consumption, relatively smaller brains, slower activities of ATPases, reduced membrane leaks) have generally all been found in most of the clades when tested for interspecific comparisons. Thus these physiological mechanisms are likely additive (or operating at different levels within the organism) rather than being alternatives. However, in general, we have much less understanding of these physiological patterns during ontogeny, even in model species, and even less information across populations.

The geometrically based answers to why hypometric metabolic scaling occurs suggest that mass-specific metabolic rates decline with size because larger animals face constraints that limit ATP supply or demand. Different types of constraints on larger animals have been hypothesized. Fractal Network Theory hypothesizes that transport networks limit resource supply at larger size (West et al. 1997; West et al. 1998). Dynamic Energy Budget theory suggests that the declining surface-to-volume ratios constrain nutrient uptake, limiting ATP demand (Kooijman 1986; Patterson 1992; Kooijman 2010; White et al. 2011). For endotherms, declining surface-to-volume ratios may constrain heat loss rates, constraining metabolic heat production in larger animals (Bejan 2001; Speakman 2010; Kwak et al. 2016). During locomotion, increased body masses relative to muscle and bone cross-sectional areas require compensatory changes in morphology and mechanical power output to prevent damage, thereby reducing metabolic rate (Biewener 1989; Norberg and Aldrin 2010). Such constraint hypotheses imply that smaller animals are less constrained and so are able to increase metabolic and functional rates toward some limit imposed by space availability and/or the maximal catalytic activity of proteins. Geometrically based constraint hypotheses are generally applicable to ontogenetic, static, and interspecific scaling.

Conversely, the drift barrier hypothesis proposes that the decline in intrinsic growth and mass-specific metabolic rates observed in animals is due to the population genetic environment. Effective population size declines approximately with mass^–0.2^, and this, along with longer generation times, leads to decreased natural selection and increased genetic drift in larger animals. Direct measures of accumulation of deleterious mutations over time in the lab show that the log number of deleterious mutations per genome increases linearly as log effective population size decreases. Thus larger organisms with smaller effective population sizes are more likely to be burdened by deleterious mutations. Genome size also increases in larger organisms, likely because nonfunctional genetic inserts are culled by natural selection in small procaryotes with high effective population sizes, while such inserts into the genome of larger animals may have low fitness costs and so accumulate. Maximum growth rates, like effective population size, scale with mass^0.8^, suggesting that at least maximal mass-specific growth rates, and plausibly metabolic rates, decline in larger animals due to accumulation of deleterious mutations too mild to be effectively targeted by selection. Therefore, according to the drift barrier hypothesis, hypometric metabolic scaling is due to a reduced ability of natural selection to cull mildly costly mutations as effective population sizes decline in larger animals. However, the drift barrier hypothesis is not likely applicable to ontogenetic or static scaling patterns.

Social synergy hypotheses arise from studies showing hypometric metabolic scaling in social insects and other colonial organisms. Social synergies among cells within organisms or among individuals within groups may increase energetic efficiency through mechanisms, such as division of labor. Larger organisms or colonies may have increased capacities to specialize cells or individuals for tasks, increasing their efficiency and decreasing mass-specific costs of task performance (Fewell and Harrison 2016).

Biotic interaction hypotheses suggest that animals of different sizes experience different natural selection forces, driving hypometric scaling, rather than focusing on the challenges of large size. These hypotheses focus on the idea that the size of an individual affects the fitness landscape for traits such as locomotion, neural activity and growth, with smaller animals experiencing more selection for performance and larger animals experiencing more selection for safety (Harrison 2017; Kozłowski et al. 2020). These biotic interaction hypotheses usually invoke trade-offs. Trade-offs assume that total energy allocation to processes, such as survival, growth, and reproduction is limited, and therefore allocation to each of these is the result of selection that maximizes overall fitness (Stearns 1989; Kozłowski et al. 2020).

The biotic interaction hypotheses incorporate the idea of correlational selection. Correlational selection occurs when the expected fitness values for one trait depend on values for another trait (Svensson et al. 2021). Mechanistically, hypometric scaling patterns may have arisen as a direct result of underlying microevolutionary processes of correlational selection between mass and metabolic rate (Arnold et al. 2001; Roff and Fairbairn 2012). Within species, there are cases showing metabolic rate and body mass to be both under selection and heritable (Pettersen et al. 2018a). Body sizes of mammal species tend to be quite consistent through geological time, except during periods of major environmental change (Smith et al. 2004). Recent evidence supports the presence of persistent multivariate selection and a strong positive genetic correlation between mass and metabolic rate in insects, birds, and mammals (White et al. 2019; Beaman et al. 2020) Hence, interspecific hypometric scaling may emerge as a consequence of selection acting within species to maximize lifetime reproduction (Kozlowski and Weiner 1997). Laboratory and field studies have supported the presence of correlational selection acting on combinations of mass and metabolic rates (Johnston et al. 2007; Artacho et al. 2015; Bartheld et al. 2015; Schuster et al. 2021); however, formal estimates of selection are exceedingly rare. These traits are hypothesized to be under context-dependent selection, whereby biotic and abiotic factors can change the strength and even direction of selection, potentially explaining variation in metabolic scaling observed across clades and environments (Nilsson and Nilsson 2016; Williams et al. 2016; Pettersen et al. 2020). Explaining how correlational selection acting within species may have shaped metabolic scaling interspecifically across the tree of life remains a key challenge of metabolic ecology.

Context-specific effects on body size and metabolism are becoming well-documented both during ontogeny and across populations. For example, better resource conditions during ontogeny can be associated with larger adult body size, higher energy content, and greater expression of sexually selected traits (Glass and Stahlschmidt 2019; Gershman et al. 2022; Xu and Fincke 2022), suggestive of patterns similar to those observed across species. As an example of context-specific variation in the evolution of body size and metabolic scaling, Moffett et al. (Moffett et al. 2022) has examined how metabolic scaling differs in populations that have recently invaded different thermal environments. The freshwater fish *Gambusia affins* (Western mosquitofish) inhabits isolated ponds and streams covering a broad thermal gradient (19–37°C) in both California and New Zealand, in which they have adapted for ∼180 generations. Across these ponds, this fish shows multiple adaptations to cope with warming; including smaller body sizes and increased reproductive rates (Fryxell et al. 2020) and increased allometric scaling exponents with warming. Metabolic scaling exponents increased from 0.70 to 0.87 with increasing habitat temperature, reducing the energetic penalty for being small, potentially aiding in fitness of the warm-adapted populations (Moffett et al. 2018). Temperature effects were stronger on pregnant females, who exhibited steeper scaling coefficients than males (Moffett et al. 2022).

One possibility is that constraint and trade-off mechanisms both exist and interact, but that their effects predominate at different scales. For example, it seems implausible that effects of declining surface-to-volume ratios can have no effect on the capacities of exoskeletons and muscles to resist breakage during locomotion across the many orders of magnitude change in body size existing in all terrestrial environments. It is generally accepted that terrestrial endotherms cannot be smaller than a few grams in body size due to limitations on mass-specific heat production (McNab 1983; Smith et al. 2004). Therefore, some constraints of the physical environment imposed by animal geometry seem extremely likely. However, over smaller size ranges, as might occur intraspecifically or among related species, it seems reasonable that such constraints may be overcome and that biotic interactions may be driving observed scaling patterns.

## Evolution of hypometric metabolic scaling: the need to connect intra- and interspecific patterns

While the best-documented and most consistent patterns of metabolic scaling occur across large ranges of body sizes across species, hypometric metabolic scaling is often observed within species as well, during ontogeny and across different individuals of the same life stage. These intraspecific variations in mass and individual properties provide the grist for the mill of the evolutionary processes that produce speciation. Both within and across species, metabolic scaling will depend on genetic factors, plasticity of individuals, and natural selection. Experimental designs that allow assessment of these effects that connect from ontogeny to population-level, and across related species, may provide a critical synthetic framework for understanding the evolution of metabolic rate scaling.

While hypometric scaling of metabolic rates is often observed during ontogeny, developmental patterns of metabolism and correlated traits can differ from scaling relationships seen in interspecific comparisons. Unlike with comparisons across adults, during ontogeny, ATP allocation shifts from growth toward maintenance and reproduction with age (Von Bertalanffy 1938, 1957). As another example, younger animals have higher brain:body mass ratios relative to that expected for interspecific comparisons (Eberhard and Wcislo 2011; Tsuboi et al. 2018). As another, during ontogeny, mass-specific locomotory capacities tend to increase with body mass, opposite to the pattern usually observed across species (Jayne and Bennett 1990). Further, the differing types of specializations of male and female adults (e.g., weapons vs. offspring production) suggest that comparisons of the metabolic scaling of the sexes can inform the importance of such life-history differences to the pattern of metabolic scaling. In many animal clades, larger species tend to have larger sexual dimorphism in adult body size (Rensche's rule [Blanckenhorn et al. 2007]), this sometimes leads to extreme sex-differences in interspecific body size and growth rates (Somjee et al. 2022). In general, these differences in size-associations of the key traits between ontogenetic and cross-species comparisons may be useful for testing the universality of scaling hypotheses; or alternatively, for demonstrating that different mechanisms drive hypometric metabolic scaling within vs. across species.

**Fig. 1:**
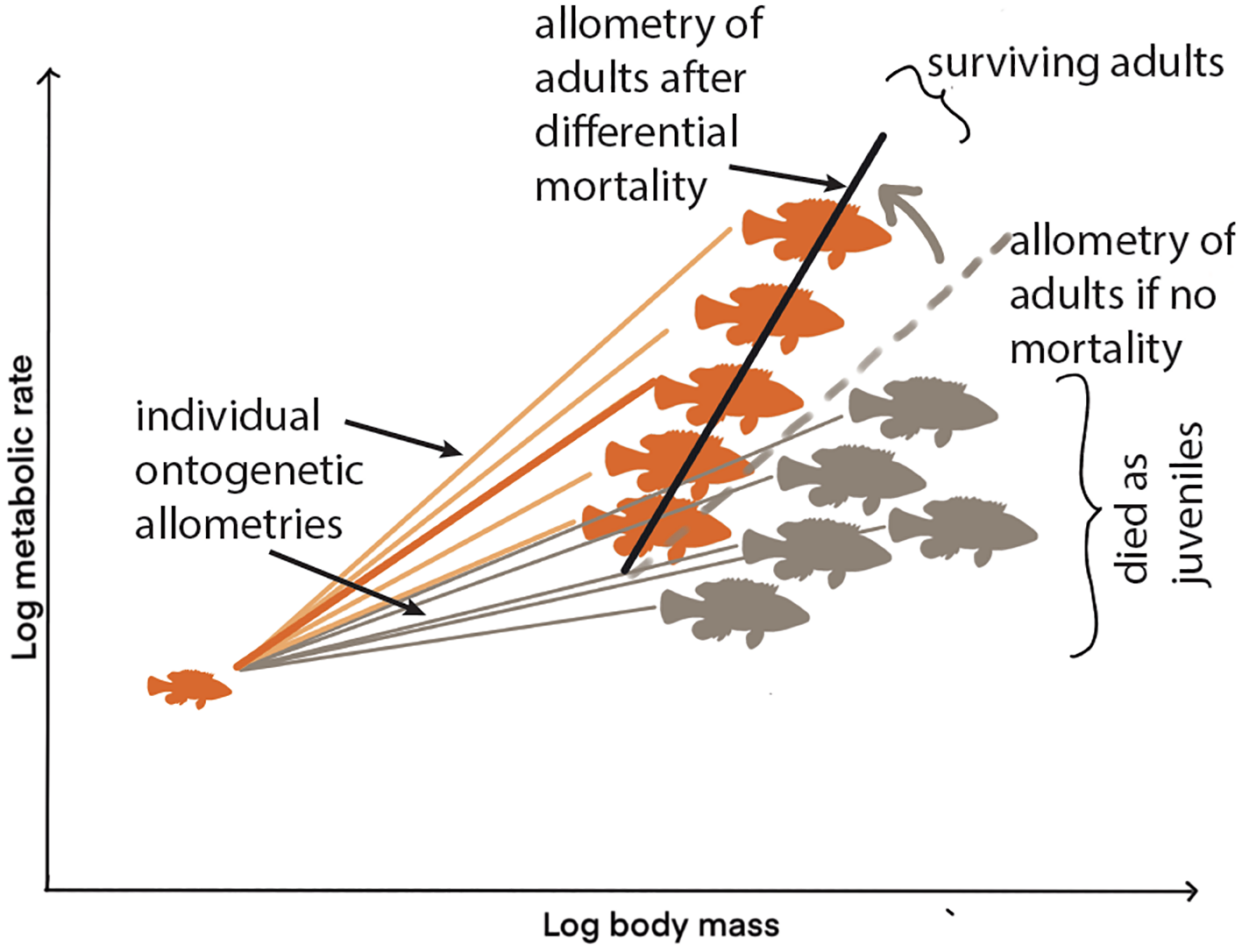
Metabolic allometric patterns of adults can differ from ontogenetic patterns for many reasons. One reason is that adult size depends on both growth rate and development time. In this example, the individuals in gray had low metabolic rates for their mass, perhaps due to low food intake and growth rate. However, they have the potential to reach the same mean mass (perhaps with longer development time), and the resultant static allometric relationship (gray dashed line) is steeper than the mean ontogenetic allometry. If all of the gray individuals die (perhaps due to predation), then the resultant static allometric relationship of the orange survivors (black line) is even steeper. Understanding the ecological and life-history causes of shifts in mean ontogenetic to static allometries have great potential for revealing natural selection effects on metabolic allometric patterns.

Many laboratory studies of body size, metabolism, and life history study animals housed individually, missing potential key biotic interactions that occur in the field that may be critical for the evolution of these parameters. Fish have higher metabolic scaling slopes when comparing across individuals relative to that observed longitudinally (Norin and Gamperl 2018; Norin 2022). Under restricted food conditions, which seem more likely to reflect field conditions, there was high variation in the metabolic scaling patterns of individual, group-reared fish, with some individuals growing strongly and showing metabolic scaling exponents near one, and other individuals showing strongly hypometric and even reductions in absolute metabolic rates at larger sizes, likely due to strong food deprivation. Across fish species, resting metabolic scaling slopes approach 1. Norin (Norin 2022) suggests that this high metabolic scaling slope may result from natural selection, with fish successful in achieving high growth and metabolic rates being most likely to survive and so determining the static allometry pattern (Fig. 1). Glazier has directly shown the role of size-specific predation in altering the metabolic scaling exponent, in this case in an opposite direction, with more predation on larger individuals (Glazier and Paul 2017). Together, these studies suggest that studies that examine metabolic scaling at ontogenetic, static, and evolutionary level, in a natural ecological context, have great potential for aiding our understanding of the evolution of metabolic scaling.

## The need for better field data on life-history variables related to metabolic rate

The equal fitness paradigm (EFP: Brown et al. 2018; updated and extended: Burger et al. 2019, 2021; Brown et al. 2022) provides a biophysical foundation for the central importance of energy in life history, ecology, and evolution. With stable populations and biodiversity, all species regardless of size are equally fit, because parents transfer equal quantities of energy and biomass to surviving offspring in the next generation. The seminal equation is

(1) $$\begin{equation*}
{\rm{\mathit{ E} = }}{{\rm{\mathit{ P}}}_{{\rm{coh}}}}{\rm{\mathit{ GFQ}}},
\end{equation*}$$

where *E* is energetic fitness, *P*_coh_ is the mass-specific rate of biomass of production over one generation, *G* is generation time (average lifespan at age of reproduction), *F* is the fraction of production that is passed through to surviving offspring (the fraction [1 − *F*] is lost to pre-reproductive mortality and left in the ecosystem), and *Q* is the near-constant energy density of biomass (∼22.4 kJ g^-1^). The EFP was originally inspired by the theoretical assumption of limited energy resources and the empirical demonstration of equal-but-opposite scaling of production rate and generation time with body mass (so the product, P*G is invariant; Brown et al. 2018). In a steady-state world (unchanging abundances, body sizes, and species diversity), *E* is the same for all species, because on average parents exactly replace themselves with an equal energy content, biomass, and number of surviving offspring each generation.

The EFP addresses metabolic scaling only indirectly. Equation 1 contains only the values—not any scaling—of production rate and generation time. However, decades of research have shown that these and many other biological rates and times tend to be closely correlated with body mass (e.g., Peters 1983; Sibly et al. 2012). Smaller animals with shorter generation times generally assimilate and use energy at faster rates (per unit mass) than larger animals with longer generation times. The EFP does not offer an explicit testable hypothesis for such scaling patterns. It does, however, imply that *G* = 1/*P* is the fundamental pace of life, set by natural selection in response to interactions among species in ecological communities.

Most studies of metabolic scaling have used laboratory data, but Equation 1 and all biotic interaction hypotheses focus on interactions in the field. Therefore, priorities for empirical research should include more and better data on *P*_coh_, *G*, and *F* in the field (Burger et al. 2021). Comparisons of wild, managed, and domesticated animal populations, and artificial systems in which species can evolve in competition with each other will be useful for evaluating biotic interaction hypotheses and the EFP. For example, can competition change metabolic scaling, due to either links with production rate or efficiency? In such artificial communities, can animals evolve to have both longer generation times and higher production rates than competitors?

## Approaches to testing ultimate hypotheses for hypometric metabolic scaling

As there are many hypotheses for hypometric scaling of metabolic rate (Table 2), and many physiological and life history correlates of body size (Table 1), a general fit of a model to metabolic and life-history parameters is insufficient to prove causality (Kearney and White 2012; Harrison 2018; Kozłowski et al. 2020). The challenge, then, is how to test particular hypotheses of hypometric metabolic scaling. For within-species studies, we can manipulate biotic and abiotic conditions, and/or use artificial selection to test predictions from the various ultimate hypotheses. Across-species, as for paleontology and economics, we can use comparative studies (natural experiments), across space or time. Many such approaches are described in the papers in this volume; other ideas are described below. It should be remembered, however, that with complex biological phenomena, a single test is unlikely to be definitive, and strong inference approaches that consider all the relevant data are likely to be necessary (Platt 1964; Fudge 2014). As the proximate questions are increasingly resolved, at least across species, and the ultimate questions are the most controversial, we focus below on possible approaches to test the various ultimate hypotheses for hypometric metabolic scaling.

## Testing ultimate hypotheses for hypometric metabolic scaling

### Geometric-based hypotheses

#### Heat balance hypothesis

At least in homeothermic endotherms, declining surface-to-volume ratios with size, and the need to match heat production to heat loss for thermoregulation, causes hypometric scaling of resting metabolic rate. This hypothesis can be viewed as a constraint hypothesis (larger animals are more challenged to eliminate excess heat (Speakman 2010), or as a simple consequence of geometry (Rubner 1883).

In support of this hypothesis, marine mammals have higher metabolic rates than similarly sized terrestrial mammals, matching their greater heat loss due to the higher thermal conductivity and specific heat of water relative to air (Williams 2022). Also, the scaling exponent of marine mammals is 0.69 (Williams 2022), near the predicted value based on surface-to-volume ratios. The observation that mitochondrial leak rates are higher in smaller mammals, and in mammals in marine environments, provides strong evidence for selection on cell and organelle properties to allow matching of heat production to heat loss (Wright et al. 2021). Further tests on endothermic populations with recent evolutionary history of body size and/or changes in thermal environment could further support this hypothesis. Genomic studies that confirm size-related selection on these leak-related proteins could also strengthen support for the hypothesis that geometric effects on heat balance cause metabolic scaling in endotherms.

As noted above, in addition to simple matching of heat production to heat loss, larger endotherms may be more constrained by heat loss when faced with warm conditions due to their reduced surface-to-volume ratios, selecting for reduced metabolic heat production rates and associated processes that require energy (Speakman 2010). Multiple studies have provided evidence that mammals can be limited in various measures of performance (growth and lactation rates) at high temperatures (Speakman 2010). However, it is not yet clear how heat-susceptibility scales with body size in endotherms. Some studies with birds suggest that smaller birds are more heat-sensitive, perhaps because their greater surface-to-volume ratios cause them to exchange heat more quickly (Smith et al. 2017). Tests of the scaling of pelage thickness and heat dumping capacities (maximal thermal conductance) could test this hypothesis, as well as more tests of the scaling of heat-sensitivity in endotherms (Harrison 2018).

Paleontological data across clades could also contribute to testing heat balance hypotheses for metabolic scaling and its interactions with selective forces that change body size in endotherms. As currently found across latitudes (Bergman's rule), mammals and birds increased in size as temperature declined through the Cenozoic, supporting the hypothesis that larger endotherm body sizes are favored under colder conditions (Smith et al.1995, 2010; Secord et al. 2012; Saarinen et al. 2014). If ectothermic vertebrates do not show this pattern, this will support the hypothesis that the size changes observed in endotherms results from the advantages related to large body size (lower heat loss rates and better cold-resistance) in the cold. Indices of metabolic rates of these fossil endotherms (e.g., bone capillarity) could determine whether larger size was associated with lower mass-specific metabolic rates.

The evidence is strong that endothermic vertebrates usually match resting heat production to heat loss in a size- and environment-related manner, consistent with a heat balance hypothesis for hypometric scaling of basal metabolic rates in these clades. Mechanistically, the hypometric scaling of metabolic rate, differences associated with the environment (terrestrial vs. aquatic), and the higher metabolic rates of endotherms vs. ectotherms are at least partially explained by the scaling of mitochondrial proton leak. Smaller mammals and birds generate more heat per gram at rest than larger ones at least partially by having higher mitochondrial proton leak rates, thus reducing the efficiency with which food is converted to ATP (Porter and Brand 1995a; Porter et al. 1996; Mélanie et al. 2019). The hypothesis that this high leak rate has been selected for heat generation of resting mammals and birds is supported by the fact that leak rates and efficiencies of conversion of oxygen and fuel to ATP is size-independent when mammal mitochondria are operating near maximal (Mélanie et al. 2019). While data for ectotherms are limited, it appears that mitochondrial proton leak is not higher in smaller ectotherms (Hulbert et al. 2002; Polymeropoulos et al. 2017), supporting the hypothesis that geometrically driven effects on heat balance may be the evolutionary cause of hypometric scaling of resting metabolic rates in endotherms but not ectotherms.

#### Efficient rates of transport through circulatory systems decline

(Fractal Network Theory/Metabolic Theory of Ecology [West et al. 1997; West et al. 1998; West et al. 1999]). This hypothesis suggests that larger organisms experience increasing constraints on resource transport through internal networks, causing associated declines in energy-requiring processes. This set of theories has had strong impact, forming the foundation of the Metabolic Theory of Ecology. However, as discussed in Brown (Brown et al. 2022), a challenge with fractal network theories has been the tremendous structural variation in the transport systems of animals, making it difficult to treat this as a general theory.

A variety of experimental tests of this transport hypothesis have been carried out. One important test of this theory is comparison of the metabolic rates of cells *in vivo* vs. *in vitro*, as the theory suggests that metabolism of cells *in vivo* is limited by supply. Indeed, West et al. showed that the metabolic rate of cultured cells was independent of body mass, suggesting that they were released from constraint (West et al. 2002). However, cultured cells are well-known to dedifferentiate, and other studies have found that the metabolic rate of tissues dissected from organisms have metabolic rates that scale hypometrically *in vitro* (Porter and Brand 1995b). Another way that this theory has been tested has been to look for evidence of direct limitations on transport in larger animals (Harrison 2018). Indeed, larger skin-breathing sirens (Ultsch 1974) and pychnogonids (Lane et al. 2017) are more oxygen-limited. However, safety margins for oxygen transport are size invariant across insect, fish, and mammal species, arguing against a general increase in transport limitations in larger animals, except perhaps in skin-breathers (Morrison and Rosenmann 1975; Nilsson and Östlund-Nilsson 2008; Harrison et al. 2014). Supporting this conclusion, oxygen transport capacities match maximal metabolic rates, regardless of body size (Seibel and Deutsch 2020). Another test for constraints on transport systems in larger animals is to determine whether larger animals exhibit increased evidence of biochemical, morphological, or physiological adaptations to overcome such a constraint (Harrison 2018). For example, we know that many animals living in hypoxic environments increase the surface areas of their gas exchangers and increase the density of capillary beds (Sollid and Nilsson 2006; Breen et al. 2008). In contrast, most larger animals generally have reduced density of oxygen transport structures, matching their lower mass-specific metabolic rates, arguing against oxygen transport limitations in larger animals causing the hypometric scaling of metabolic rates (Harrison 2018). Kearney and White suggested that if this hypothesis is correct, ultimate size and growth rate in animals should be directly proportional to blood oxygen levels (Kearney and White 2012). Most animals that have been tested are smaller when developing in hypoxia, but this generally occurs via hormonally mediated mechanisms rather than oxygen-limitation of metabolic rate, and hyperoxia rarely increases animal body size (Owerkowicz et al. 2009; Wang et al. 2009; Harrison et al. 2015; VandenBrooks et al. 2020). Kearney and White also suggested that this theory predicts that exercise training should reduce the scaling exponent, and that the thermal tolerance zone for aerobic respiration should be inversely related to body size (Kearney and White 2012). To our knowledge, these predictions have not yet been tested.

#### Declining body or gut surface-to-body volume ratios reduce mass-specific nutrient intake (Dynamic Energy Budget models)

This hypothesis suggests that declining surface-to-volume ratios cause larger species to be constrained by consumptive/digestive/absorptive processes, causing associated declines in energy-requiring processes (Kooijman 2010; Maino et al. 2014). Decreases in surface-to-volume ratios are also likely to affect gas, ion, and heat exchange for many animals, potentially influencing energy turnover. One argument against surface-to-volume limitation models is that metabolic rates are often found to scale with exponents nearer to 0.75 than the 0.67 predicted by surface area scaling. However, surface area scaling could cause hypometric scaling of metabolic rate, perhaps slightly ameliorated by compensatory adaptations of larger animals to raise their energy turnover and performance.

A number of experimental tests of this hypothesis have been performed. White et al. experimentally manipulated size in 2D fast-growing bryozoans and found a scaling exponent of 0.5, consistent with surface limitations on gas or nutrient exchange (White et al. 2011). If surface area constraints directly limit metabolic rate by constraining digestion or absorption across the gut in animals, we might expect to observe increased gut retention times and plausibly stronger scaling of food consumption relative to metabolic rate (which could compensate for low digestive/absorptive rates by providing more fresh food). Increased retention time in larger animals is broadly observed (Clauss et al. 2007; McWhorter et al. 2009; Müller et al. 2013). However, increased scaling of consumption relative to metabolism is observed in herbivorous but not in omnivorous or carnivorous mammals, possibly because larger mammalian herbivores are generally less selective and consume poorer quality food (Clauss et al. 2007; Clauss et al. 2013). Similarly, as described above for transport hypotheses, if gut surface area is limiting to transport in larger animals, we might expect to see compensatory adaptations such as increased microvilli or transporter density with increased body size. Four different studies in mammals and birds have found no evidence for mechanisms indicative of adaptations to compensate for low gut surface area-to-volume ratios, suggesting that constraints on gut transport are unlikely to cause hypometric scaling of metabolic rate (Lavin et al. 2008; Müller et al. 2013; Steuer et al. 2014; Price et al. 2015). Thus, data to date suggest that surface area-mediated nutrient transport might determine metabolic scaling in some marine invertebrates, but likely not in endothermic vertebrates.

Kearney and White suggested multiple insightful additional tests of these Dynamic Energy Budget-related hypotheses that, to our knowledge, have not yet been implemented (Kearney and White 2012). They suggested that limb regeneration rates should be faster than ontogenetic growth rates if growth is limited by surface area, since regeneration would presumably be supplied at least partially by internal stores. Additionally, they suggest manipulation of reserve density should change the metabolic scaling exponent, with *b* = 1 for zero reserve density and *b* < 1 if reserve density is maximal.

#### Drift barrier hypothesis

Larger animals have longer generation times, and so experience reduced selection on deleterious mutations, leading to reduced mass-specific growth rates in larger organisms, and possibly reduced mass-specific metabolic rates (Lynch 2022).

Only a few tests of this hypothesis have been performed. One might expect that accumulation of deleterious mutations might reduce efficiencies, but growth efficiency is generally thought to be constant across species varying in size (DeLong et al. 2010) and to increase with size during locomotion (Biewener and Patek 2018). There is some evidence that increased genome size is correlated with reduced basal metabolic rates in vertebrates, consistent with the drift barrier being at least part of the explanation for hypometric scaling of metabolism (Kozlowski et al. 2003; Uyeda et al. 2017).

Many additional tests for this hypothesis are possible. If correct, then larger animals should have more deleterious mutations in protein-coding genes, and enzymes with lower mass-specific catalytic capacities. Manipulations of effective population size over generations in the lab should change the load of deleterious mutation, enzyme catalytic capacities, and organismal growth and metabolic rates.

#### Declining bone and muscle area-to-body mass ratios increases risk of fragility, requiring slower movements or adjustments that reduce bone and muscle loading

Most animals move, at least during part of their life cycle. Maximal metabolic rates usually occur during locomotion, muscle-driven movement is a major component of the energy use of animals in the field, and animals that fly have higher resting metabolic rates (Nagy 1987), suggesting that energy costs during locomotion may be important to explaining metabolic scaling (Biewener and Patek 2018). Constraints on larger animals might arise because the cross-sectional areas of bones and muscles increase more slowly than mass as animals increase in size, potentially leading to increased risk of damage (Biewener 1982, 1989). Haldane's observation that, if similarly dropped, “a mouse walks away, a man is broken and a horse splashes” illustrates how increasing size raises the risks of damage caused by mechanical forces (Haldane 1985). Can compensating for such risks drive hypometric metabolic scaling?

Fundamentally all organisms face common constraints imposed by their environment, whether it be life and movement on land, in the air or in water. These physical and geochemical constraints (temperature, gravitational loading, fluid drag, oxygen availability, etc.) are often met by evolutionary convergence on the properties of metabolic and skeletal tissues. As a result, animals must support and move themselves by muscles that have similar contractile properties for force generation and length change, tendons (apodemes, resilin pads, etc.) that may operate as elastic elements to store and return energy, and skeletal tissues that share similar material properties for supporting the forces acting on an animal. Consequently, the biomechanical and physiological requirements that must be met for physical activity related to foraging, migration, mating, and predator–prey interactions will strongly influence metabolic energetics. Therefore, how biomechanical and physiological properties scale with body size will underlie the scaling of metabolic energetics during locomotion and other physical activities.

Across terrestrial mammals, metabolic rate when running at equivalent gaits (*E*) scales hypometrically with size, with a scaling coefficient, *b* = 0.7 (Heglund et al. 1982). By contrast, the mechanical work rate (*W*) to move the animal's center of mass and swing the limbs scales independently of size (*b* = 1, Heglund et al. 1982). What factors then determine the scaling of metabolic rate during locomotion?

Because skeletal and cardiac muscles consume the majority of energy during exercise, muscle energy expenditure determines much of the pattern of metabolic scaling in relation to exercise. For example, human skeletal muscles consume 80–90% of the aerobic ATP supply during moderate to vigorous activity (Zoladz 2019). This reflects the fact that skeletal muscles constitute 40–45% of total body mass in humans, and even a higher fraction (up to ∼70%) in fish (Bone 1978; Videler 1993). Consequently, the scaling of metabolic energy rate during locomotor activity likely reflects the ATP consumed by muscles to generate the force and work needed to support an animal's weight and power its movement.

A muscle's rate of energy use is determined by the ATP cost of (1) actin-myosin cross-bridge formation (∼70%) and (2) calcium cycling to activate and relax the muscle (∼30%, [Woledge et al. 1985; Rall 1985]). The total energy consumed by a muscle is therefore strongly influenced by the rate of muscle activation and the force that a muscle must generate on a per volume basis. The active volume of a muscle, in turn, is a product of the cross-sectional area of recruited muscle fibers times their fiber length (Roberts et al. 1998; Biewener and Roberts 2000).

A common contractile property of muscle is that force generation depends on the cross-sectional area of activated fibers. The cross-sectional area of terrestrial mammalian muscle fibers (*A_m_*) scales hypermetrically, with a scaling coefficient, *b* = 0.79, owing to a slight hypermetric scaling of muscle mass (*b* = 1.07) and hypometric scaling of fiber length (*b* = 0.28, [Alexander et al. 1981]). Correspondingly, size-related changes in locomotor limb posture (Biewener 1990,^2005^) significantly increase the effective mechanical advantage of limb muscles (*b* = 0.25), such that muscle force requirements (*F_m_*) to support weight and move scales hypermetrically (*b* = 0.75), allowing for nearly uniform muscle stresses ( = *F_m_*/*A_m_*) across size within terrestrial mammals (*b* = 0.04). Consequently, a similar volume of muscle is recruited to generate the forces required during each stride of locomotion, and on a mass-specific basis, the ATP cost of force generation is likely the same in terms of active muscle volume (*b* = 0). This finding is reinforced by an analysis showing a similar mass-specific metabolic cost of generating force on the ground across different sized terrestrial birds and mammals (Kram and Taylor 1990). Thus, the size-independent scaling of active muscle volume and its associated ATP cost for generating force on the ground cannot explain the observed hypometric scaling pattern of metabolic rate vs. running speed (Heglund et al. 1982).

Although active muscle volume is size-independent, the stride frequency (*S*_freq_) of quadrupedal mammals scales inversely (hypometrically) with size (*b* = −0.15) at comparable gait-related speeds (Heglund and Taylor 1988). Consequently, the combined scaling of mass-specific active muscle volume with stride frequency predicts that metabolic rate should scale with a coefficient of 0.85. However, this still leaves a gap with respect to the observed scaling coefficient (0.7) of metabolic rate during terrestrial locomotion.

It seems likely that an increase in elastic energy savings by tendons and ligaments (*U*_elas_) to reduce muscle work, relative to the mechanical work of moving the body (*W*) with increasing size, could explain the remaining difference between predicted vs. observed patterns of scaling of mass-specific metabolic rate during locomotion. In a broad comparison of skeletal muscle fiber area (*A_m_*) vs. tendon area (*A_t_*), Pollack and Shadwick found that *A_m_*/*A_t_*—a proxy for tendon stress—scaled hypermetrically (*b* = 0.17) for principal muscle extensor tendons (Pollock and Shadwick 1994). Given that elastic storage and recovery depends on tendon stress squared times tendon volume, *U*_elas_ is predicted to scale hypermetrically (*b* = 1.28). Past studies have estimated the savings of muscle work due to elastic energy recovery to be in the range of 20–50% for humans, kangaroos, wallabies, dogs, and horses (Cavagna et al. 1977; Ker et al. 1987; Biewener and Baudinette 1995; Biewener 1998); all larger species for which *U*_elas_ is predicted to scale favorably. Consequently, it seems likely that increased elastic energy recovery and decreased muscle work might well provide the additional metabolic energy savings needed to explain the observed hypometric scaling pattern of metabolic rate during locomotion. A challenge to demonstrating this is that muscles consume metabolic energy to generate force as well as to perform work, and resolving how much each contributes to overall energy cost is difficult. Further, muscles generate force, consuming metabolic energy, to store and recover elastic energy in their tendons.

Together, these data provide a proximate “how” explanation of the hypometric scaling of metabolic rates during terrestrial locomotion. Metabolic rates during locomotion scale hypometrically at least partially because maximal locomotory muscle frequency declines with size, reducing the cost of calcium and myosin-head cross-bridge cycling, and likely improving elastic energy storage. These explanations also likely apply to both flying and swimming, as similar trends in the frequency of limb cycling are observed (Greenewalt 1975; Sato et al. 2007). There are some important exceptions to the generally observed decline in muscle frequency with body size, and these seem to “prove the rule”. For example, in stingless bees, for bees heavier than 50 mg, both wingbeat frequency and the mass-specific cost of hovering increase as size declines, but as species miniaturize further into smaller size ranges, wingbeat frequency is invariant with size, and the mass-specific cost of hovering declines instead of increasing (Duell et al. 2022).

However, the ultimate explanation of this pattern is less definitive. While we have classified this hypothesis as a geometric constraint-type hypothesis, the contrasting benefits and costs of crouched-fast vs. upright-slow limbs and muscles are clear examples of trade-offs in animal design. The decline in stride frequencies in larger mammals can be explained by declining muscle and tendon area-to-body mass ratios with size, and the need to avoid mechanical damage (Biewener 1982,^1989^). However, when considering the trends going toward smaller sizes, this explanation seems incomplete. Smaller animals have a more crouched postures and faster muscles in order to generate more power in a shorter time, enabling them to attain absolute speeds near to those of larger animals despite their shorter limbs (Usherwood 2013; Biewener and Patek 2018). The crouched position and fast muscles of smaller animals seems likely to have resulted from interactions with the biotic environment, especially competition and predation. Outcomes of competition and predation depend on absolute speed, so it seems likely that smaller animals may experience greater selection for relative speed, causing the observed changes in skeletal structures and muscle properties that increase mass-specific costs in smaller animals (Harrison 2017). From this perspective, the hypometric scaling of metabolic rate during locomotion ultimately arises from selection for safe, high locomotory performance, which requires higher mass-specific cost in smaller animals.

Experimental tests of these differing ultimate explanations will require comparative approaches. For example, the hypothesis that changes in muscle properties, frequencies, and posture are driven by the need to avoid breakage implies that environments that differ in this risk should affect the pattern. Comparison of aquatic vs. terrestrial environments, or flatland vs. mountain-adapted species should reveal predictable alterations, with animals from environments posing less risk of mechanical damage having reduced evidence of protective muscle, bone, and tendon properties, and lower scaling coefficients. The hypothesis that variation in relative speed and mass-specific metabolic rate is driven by predation suggests that in toxin- or structurally protected clades with no selection on locomotory speed should not show the normal size-related changes in muscle frequency or the mass-specific rate of energy use.

### Biotic interactions hypotheses

Most of the biotic interaction hypotheses emphasize how natural selection may differentially affect aspects of organismal function as body size changes. Trade-offs occur because organisms only have so much space or resources, and so allocation of resources to one function precludes others. For example, organisms that prioritize growth rate must maximize feeding and anabolic processes and structures, necessarily leaving less material and space for structural defense. Very large brains in miniaturized species occupy space that could be used by other tissues.

Body size influences how animals interact with the biotic environment for many reasons. Without compensation, the smaller lengths of bodies, jaws/mandibles, and appendages of smaller animals lowers their absolute speed, consumption, and fighting ability, making them easier prey and poorer competitors, and increasing extrinsic mortality. Similarly, without compensation, their smaller brains and sensory organs reduce their neural capacities relative to larger animals. Higher extrinisic mortality can select for faster growth rate and earlier maturation (Stearns 1992; Gasser et al. 2000). All such trade-offs have the potential to apply to interspecific, static, and ontogenetic scaling patterns.

Examination of how ecological conditions such as temperature, population density, and predation affect metabolic scaling has demonstrated that metabolic scaling is sensitive to biotic interactions, though more research is needed. Studies of situations in which animals are released from normal ecological constraints have particular value in revealing causality. Islands, artificial selection for high growth rate or size, and conditions after mass extinctions all represent situations in which animals may be released from “normal” levels of competition and predation or transferred to new conditions of resource availability. As an example, studies of island fauna have revealed that such species tend to have lower mass-specific metabolic rates than mainland counterparts, perhaps due to lower food availability associated with high intraspecific species densities and low fertility (McNab 2002), supporting the importance of ecological factors in determining metabolic rates.

#### Neural trade-off hypothesis

Hypometric metabolic scaling occurs at least partially because of size-related selection on neural processes underpinning behavioral performance and/or cognition, with smaller animals investing more in energetically expensive brains to maintain similar behavioral/cognitive capacities as larger animals (Haller's Rule (Yopak et al. 2010; Seid et al. 2011; Coto and Traniello 2021). This is a component of the performance-safety trade-off hypothesis (Harrison 2017).

Brain size scales hypometrically with respect to body size across diverse animal clades (Eberhard and Wcislo 2011; Polilov and Makarova 2017; Burger et al. 2019; Godfrey et al. 2021; McCurry et al. 2021), with few exceptions (e.g., Groothuis and Smid 2017). If increased total brain or brain compartment size allows increased information processing ability to meet complex behavior/cognitive demands (Chittka and Niven 2009; Dunbar and Shultz 2017; DeCasien and Higham 2019; Kilmer and Rodríguez 2019; ), then selection for neural performance may drive the evolution of increased relative brain investment in smaller animals to maintain similar behavioral/cognitive capacities during predator avoidance or competition with larger animals (Seid et al. 2011; Coto and Traniello 2021; Muratore et al. 2022; Coto and Traniello 2022). One potential mechanism linking brain and body size scaling may be genes that simultaneously influence brain and whole-body growth, with greater expression early in life or in smaller species (Riska and Atchley 1985).

Hypometric brain size scaling is expected to contribute to hypometric scaling of whole body metabolic rates if brains are expensive, exhibiting higher metabolic costs than other organs. However, brain metabolic rate is usually <8% of whole-body metabolic rate (Mink et al. 1981; Kern 1985; Rolfe and Brown 1997; Karbowski 2007; Bordone et al. 2019), suggesting that effects of brain scaling on whole body metabolism may be small. Furthermore, brain miniaturization is likely facilitated by adaptive changes in neuron function and structure and/or molecular mechanisms that maximize energy efficiency (Niven and Laughlin 2008; Niven and Farris 2012; Kamhi et al. 2016). Yet, in miniaturized invertebrates in which the brain may compose up to 15% of body size (Seid et al. 2011; Polilov and Makarova 2017) and brain size increase is accommodated by neuropil extension into the thorax or leg segments (Quesada et al. 2021), it seems likely that brains may contribute very significantly to whole-body metabolism. In general, we are lacking data on the metabolic scaling of brains to test this hypothesis in invertebrates.

One experimental test of the importance of the neural hypothesis in driving hypometric metabolic scaling is comparison of clades with and without brains, though these comparisons have many complicating factors, such as evolutionary history, life-history differences, and correlations with other systems, such as muscles. According to some studies, plants and prokaryotes do not exhibit hypometric metabolic scaling (Reich et al. 2006; DeLong et al. 2010), supporting the importance of neural and/or locomotory systems to hypometric metabolic scaling, though other studies suggest plant metabolic rate does scale with an exponent <1 (Hatton et al. 2019).

An additional test of the neural hypothesis will be to examine the behavioral capabilities of small and large animals, in particular, constraints on behavior imposed by small brain size. If smaller animals are selected to maintain high behavioral capabilities, we hypothesize that behavioral/cognitive capabilities will not be significantly affected by the brain size, and that there will be substantial evidence that the nervous systems of smaller animals will be adapted to achieve high mass-specific function. As yet, there have been few tests of the behavioral capacities of small and large animals, but data suggest that small animals are generally similar to large animals in behavioral capacities (Chittka and Niven 2009), though perhaps not duration of memory (Kilmer and Rodríguez 2019). Artificial selection experiments have provided mixed results for the impact of increased relative brain size on cognitive abilities (Kotrschal et al. 2013; van der Woude et al. 2019). These studies suggest that relative brain size may impact some behaviors more strongly than others and these associations may be clade-specific.

Another class of experimental tests will be to determine whether brains of smaller animals exhibit structural and functional traits consistent with greater selection to preserve mass-specific function, and whether this elevates mass-specific cost. Smaller mammals (Herculano-Houzel 2011) and hymenopterans have higher brain cell densities (Godfrey et al. 2021), as expected if smaller animals are selected to increase mass-specific neural performance. Higher limb cycling frequency in smaller animals suggests a need for faster temporal resolution of information, which seems likely to increase mass-specific costs. Comparative studies of how body size influences the properties of neurons and glial cells will be useful to understand how size affects neuronal system function.

#### Developmental and growth rate trade-off hypotheses

According to this hypothesis, the higher extrinsic mortality, and/or use of temporally or spatially small niches, of smaller species causes them to be more strongly selected for high mass-specific growth and developmental rates, and thus shorter time to maturity. Higher mass-specific growth and developmental rates can drive higher mass-specific metabolic rates in smaller animals due to the high costs of foraging, digestion, absorption, and anabolism. Conversely, large species with reduced predation pressure and a greater need to survive through poor resource conditions may have reduced selection for high growth rates, lowering mass-specific metabolic rates.

All metazoans must develop from a fertilized cell through juveniles stages to adulthood. Embryonic and juvenile periods are energetically costly and also subject to high mortality (Houde and Hoyt 1987). Given that the completion of development is a crucial bottleneck for reaching reproductive maturity, there is likely selection for fast completion of embryonic and juvenile development—to build feeding structures and reach a size refuge sooner, reducing the risk of predation and starvation (Prasad et al. 2001). Previous work has demonstrated that both within and among species, the time from fertilization to nutritional independence often increases with initial offspring size—that is, smaller offspring develop faster than larger offspring. However, while small offspring develop faster, they also use energy less efficiently than large offspring and complete development with a lower proportion of their initial reserves (Pettersen et al. 2018b). Metabolic rates of embryos scale hypometrically with offspring size both within and among species (Pettersen et al. 2022).

While the mechanisms underlying offspring size effects remain unclear, size-dependent developmental rates may have important consequences for the evolution of offspring size and metabolic rate, and thus the maintenance of hypometric scaling more generally. In environments where mortality during development is high (e.g., high predation or competition), there may be selection for fast developing, small offspring, with high-mass specific metabolic rates. The strength and direction of selection on offspring size is highly context dependent however, and environmental heterogeneity is likely to maintain variation in metabolic phenotypes (McDonald and Ayala 1974; Pettersen et al. 2020). For example, large offspring that are slow-developing with low mass-specific metabolic rates may be at an advantage when predation and competition is low, while small, fast-developing offspring with high mass-specific metabolic rates will reach a size refuge sooner and avoid predation (Auer et al. 2018).

Experimental tests of the relationships between fitness, metabolic rate, and growth rate can be performed by varying the environment. For example, variation in food availability has been shown to alter ontogenetic and static scaling of metabolism in fish. When food is plentiful and predictable, there is a positive relationship between growth rate and standard metabolic rate (i.e., individuals with relatively high standard metabolic rates for their size grow faster than low-standard metabolic rate conspecifics (Metcalfe et al. 2016; Norin et al. 2016). However, when food is limited or unpredictable, standard metabolic rate and growth rate are negatively correlated or uncorrelated (Norin and Malte 2011; Hoogenboom et al. 2012; Reid et al. 2012). Variation in food availability thus causes variation among individuals in the relationship between standard metabolic rate and growth rate, and individuals change their standard metabolic rate to different extents when food availability changes (Auer et al. 2015). The consequence for scaling of standard metabolic rate appears to be that, in a food limited environment, individuals that initially have relatively high standard metabolic rates grow slower and gain less body mass relatively to their faster-growing conspecifics, causing low or even negative scaling exponents for those slow-growing individuals and an overall positive relationship between individual scaling exponents for standard metabolic and growth rates (Norin 2022). This not only results in highly variable scaling exponents among individuals, but also a significantly lower mean ontogenetic (within-individual) than static (among-individual) scaling exponent for standard metabolic rate (Fig. 1). These findings suggest that there is no metabolic phenotype that performs best across all conditions, but rather that environmental variability can maintain considerable variation in metabolism and metabolic scaling intraspecifically (Auer et al. 2015), and that natural selection can quickly change metabolic scaling exponents Fig. 1 (Norin 2022).

Another type of test of the role of growth and developmental rates in determining metabolic scaling patterns is to compare populations that differ in traits expected to alter parameters such as predation, competition, and food availability. For example, among conspecific populations of amphipod crustaceans, populations that experience high size-selective mortality in the field, where large individuals are preferentially eaten by fish predators, exhibit shallower metabolic scaling, and stronger shifts toward reproductive vs. somatic growth with age/size; consistent with the hypothesis that selection for high early growth rate raises mass-specific metabolic rates of younger/smaller individuals (Glazier et al. 2011; Glazier et al. 2020). Patterns of growth rates between cephalopods and fish in benthic vs. pelagic environments can explain variation in metabolic scaling coefficients (Tan et al. 2019). Moreover, fish taxa that experience higher natural larval mortality rates and, presumably, a stronger size-selective pressure for fast ontogenetic growth in early life (Sogard 1997) tend to have steeper scaling of standard metabolic rate(SMR)(Norin 2022). These results support the hypothesis that natural selection on slow growth at the individual level may result in shallow metabolic scaling for the species or across species with similar size-selective mortality rates, and *vice versa* for selection for fast growth.

#### Sexual selection hypothesis

At least during ontogeny and within populations, sexual selection contributes to hypometric scaling of metabolic rate by selecting for reproductive structures with low cost. Males and females of the same species are often distinguished by their differential investment in reproduction, but the sexes often differ in body size, growth rates, feeding ecology, territoriality, and multiple other life-history and physiological variables. These differences often lead to sex-differences in the scaling of metabolic rate. However, relatively few studies examine how different selective pressures experienced by males and females lead to differences in metabolic rates.

Sexual selection drives some of the largest differences in males and females, and sex-specific selection often pushes metabolically important physiological changes to their limits. Because sexual selection is a form of social selection, this form of selection is often directional rather than stabilizing. In other words, sexual selection drives traits to be relatively larger, and not necessarily of a shared optimal size.

Examining sex differences in metabolic rates can be valuable for understanding the scaling of metabolic rates for multiple reasons. First, because males and females share most of the same genome, and there may be opposing selective pressures on males relative to females, examination of sex-specific metabolic scaling patterns can help us to understand the effects of different selective pressures on a shared genome. Second, sexual selection provides examples of some of the largest and most extreme traits in nature. Thus, sexually selected traits and behavior provide a natural experiment where often only a single sex is selected for specific maximal metabolic rates (due to growth rates/mating displays). Therefore, examination of sex-specific metabolic scaling offers opportunities to examine links between behavioral, morphological and physiological traits, and metabolic scaling. If sexual selection contributes to hypometric metabolic scaling, then males with large, sexually selected structures should have different allometric scaling for metabolism than females (Somjee 2022). Also, in some cases, direct experiments such as removal of ornaments/weapons can be performed. This can be done while the ornaments/weapons are growing, or after maturity. Such experiments can provide a direct measure of costs of maintenance of sexually selected ornaments and weapons, and test the importance of these to metabolic scaling patterns.

#### Social synergies hypothesis

Larger, more complex groups can achieve higher energetic efficiency due to increased division of labor, leading to more specialization and efficiency of task performance (Holbrook et al. 2011; Holbrook et al. 2013b; Fewell and Harrison 2016). Such social synergies could conceivably also occur within animals, as larger animals may have a greater capacity for division of labor among cells.

Colony metabolic rate has often been found to scale hypometrically with colony size in social insects, and experimental manipulations of colony size demonstrate that colony size causally affects metabolic rate (Hou et al. 2010; Shik 2010; Waters et al. 2010; Fewell and Harrison 2016; Waters et al. 2017). Many of the mechanisms that have been proposed to cause hypometric metabolic scaling in animals seem unlikely to apply to most social insect colonies, such as heat balance, oxygen transport, or nutrient delivery, since most experiments have been done in the lab with colonies with ad lib food and in containers that ensure oxygen availability to the center of the colony. In some polymorphic species, this hypometric metabolic scaling may be driven by an increasing proportion of large (defensive) workers that have reduced mass-specific metabolic rates (Shik 2010). Within species, larger colonies with lower mass-specific metabolic rates have lower per capita activity levels, but this does not completely account for the metabolic differences (Waters et al. 2017). As suggested by the performance-safety trade-off hypothesis for animals, it may be more critical for smaller colonies to invest in expensive, high growth rates, with larger colonies able to allocate workers to less-active tasks that may aid safety (Holbrook et al. 2013a).

More data are needed on the extent to which social synergies can allow reduced whole-society or organismal cost. Additionally, in most cases, we are lacking field data on the scaling of productivity and metabolism for social colonies, hampering our ability to interpret these patterns in a natural selection context. Additionally, there are currently no data on the ontogeny of metabolic rates in social insect colonies, or studies of how ecological context affects colonial metabolism.

## Conclusions

The inter-relationship between the suite of factors affecting metabolic rate and scaling makes this discipline central to many ecological and evolutionary models. Understanding this “rule of life” is key for developing predictive models of evolution of form, community function, conservation, and how communities will be altered by climate change. These considerations suggest that development of agreed-upon models of hypometric scaling of metabolic rates should be a high priority for biology.

One major suggestion for the field is that more studies approach hypometric scaling with a perspective of testing multiple hypotheses. Far too often, papers in the field evaluate their data only with respect to one hypothesis, rather than considering how their results match or reject other hypotheses for metabolic scaling. A related suggestion is that investigators clarify whether their hypotheses address proximate or ultimate questions, and the conditions (clade, size range, and ecological conditions) to which their hypotheses likely apply.

A second major suggestion is that the field follow the suggestions of Glazier (Glazier 2005) to examine the context-dependence of metabolic scaling, including phylogenetic, biotic, and abiotic context. Such comparisons have considerable potential to determine how universal scaling patterns are, and to test predictions of specific hypotheses.

Third, to rigorously understand the evolution of hypometric metabolic scaling, more studies are needed that link ontogenetic, static, and evolutionary allometries. The generally high mortality rates of juveniles and the constraints of development mean that the traits of embryos and juveniles are likely under strong selection. How juvenile traits link to adult traits is little-studied but critical for understanding the evolution of static allometric patterns. Similarly, understanding interspecific patterns will require more data on how and why metabolic rates and correlated traits vary within species, and the ecological conditions that influence such patterns.

A fourth major suggestion is that we need much improved field data on metabolic rates and correlated variables. Quality control can be especially problematic due to the variety of methods used to assess metabolism in vastly different organisms. Inconsistencies in terminology, temporal scales, and physiological states of the organisms exacerbate the problem. Even seemingly basic information such as growth rates, maximum age, and reproduction time relative to animal mass can be difficult to collect in the field. However, all ultimate questions regarding the scaling of metabolism and its associated variables are framed in the context of the natural world. A renewed investment in understanding the natural history of animal processes is critical for progress on this central question of biology.

## Data Availability

All data are available at Dryad: doi:10.5061/dryad.g1jwstqtf.

## Notes

From the symposium “Causal mechanisms of interspecific metabolic scaling patterns’’ presented at the annual meeting of the Society for Integrative and Comparative Biology virtual annual meeting, January 3–February 28, 2022.
